# Diagnostic ability of maximum blink interval together with Japanese version of Ocular Surface Disease Index score for dry eye disease

**DOI:** 10.1038/s41598-020-75193-4

**Published:** 2020-10-22

**Authors:** Kunihiko Hirosawa, Takenori Inomata, Jaemyoung Sung, Masahiro Nakamura, Yuichi Okumura, Akie Midorikawa-Inomata, Maria Miura, Kenta Fujio, Yasutsugu Akasaki, Keiichi Fujimoto, Jun Zhu, Atsuko Eguchi, Ken Nagino, Mizu Kuwahara, Hurramhon Shokirova, Ai Yanagawa, Akira Murakami

**Affiliations:** 1 Department of Ophthalmology, Juntendo University Graduate School of Medicine, 3-1-3 Hongo, Bunkyo-ku, Tokyo, 1138421 Japan; 2 Department of Digital Medicine, Juntendo University Graduate School of Medicine, Tokyo, 1138421 Japan; 3 Department of Ophthalmology, Juntendo University Faculty of Medicine, Tokyo, 1130033 Japan; 4 Department of Strategic Operating Room Management and Improvement, Juntendo University Graduate School of Medicine, Tokyo, 1138421 Japan; 5 Department of Hospital Administration, Juntendo University Graduate School of Medicine, Tokyo, 1138421 Japan; 6 Department of Bioengineering, Graduate School of Engineering, Precision Health, The University of Tokyo, Tokyo, 1130033 Japan; 7 Department of Ophthalmology, Subei People’s Hospital Affiliated to Yangzhou University, Yangzhou, 225001 Jiangsu China

**Keywords:** Diagnostic markers, Corneal diseases, Lacrimal apparatus diseases

## Abstract

Various symptoms of the dry eye disease (DED) interfere with the quality of life and reduce work productivity. Therefore, screening, prevention, and treatment of DED are important. We aimed to investigate the potential diagnostic ability of the maximum blink interval (MBI) (the length of time participants could keep their eyes open) with disease-specific questionnaire for DED. This cross-sectional study included 365 patients (252 with DED and 113 without DED) recruited between September 2017 and December 2019. Discriminant validity was assessed by comparing the non-DED and DED groups based on the MBI with a Japanese version of the Ocular Surface Disease Index (J-OSDI) and tear film breakup time (TFBUT) with J-OSDI classifications. The MBI with J-OSDI showed good discriminant validity by known-group comparisons. The positive and predictive values of MBI with J-OSDI were 96.0% (190/198 individuals) and 37.1% (62/167 individuals), respectively. The area under the receiver operating characteristic curve (AUC) of MBI with J-OSDI was 0.938 (95% confidence interval 0.904–0.971), the sensitivity was 75.4% (190/252 individuals), and the specificity was 92.9% (105/113 individuals), which are similar to the diagnostic ability of TFBUT with J-OSDI (AUC 0.954). In conclusion, MBI with J-OSDI may be a simple, non-invasive screening test for DED.

## Introduction

Dry eye disease (DED) is one of the most common eye diseases, affecting 5–50% of the population worldwide^1^. DED causes various symptoms that decrease quality of life and work productivity^2,3^. However, DEDs may be overlooked due to their diverse and heterogeneric symptoms other than dryness. A current smartphone application-based study identified that many individuals with DED symptoms remain undiagnosed and suffer the symptoms^4–7^. Because DED is a symptomatic treatment-oriented disease, early detection and treatment are important to maintain quality of life^8,9^. Therefore, simple biomarkers are necessary for early diagnosis.

DED is a relatively new disease, proposed in 1995^10^, and the definition and diagnosis of DED have evolved over time^11,12^. In recent years, the Tear Film and Ocular Surface Society and Asia Dry Eye Society (ADES) have proposed the pathogenesis and classification of DED^13,14^. DED diagnosis was primarily performed by subjective symptom assessment, risk factor analysis, and dry eye examinations including tear film breakup time (TFBUT), tear volume, and kerato-conjunctival staining^14,15^. However, many problems are encountered in the diagnosis of DED such as the variability of dry eye examinations among examiners, and the discrepancy between subjective symptoms of DED and the value of other dry eye examinations^16,17^.

DED is becoming more prevalent due to aging society, increased digital work, and stressful society^4,6,18–20^. In addition, not only the elderly, but also young people are expected to suffer more from DED due to the increase in digital work associated with the advent of computers and smartphones^4,5^. In this context, developing a simple screening and self-check method for DED is important for self-management of the disease. Previously, we have developed the maximum blink interval (MBI) as a simple screening method for DED^21^. MBI is positively correlated with TFBUT and is useful because it can be performed by anyone, anywhere. The diagnostic criteria of DED based on the Tear Film and Ocular Surface Society and ADES share subjective symptoms and lower TFBUT as the primary DED test, and if TFBUT can be replaced by MBI, a remote diagnosis and self-monitoring app using disease-specific questionnaire, such as the Ocular Surface Disease Index (OSDI)^22^ and MBI, may be possible. In this study, we aimed to determine the potential diagnostic ability of the combination of MBI and OSDI for DED.

## Results

### Characteristics of participants

We enrolled 365 participants in this study. Table 1 shows the characteristics of the participants. All participants underwent complete examination and were eligible for analysis. The average age was 60.4 ± 16.3 years, and 85.2% (311/365 individuals) of the participants were women. Based on the ADES diagnostic criteria^14^, 113 participants were diagnosed as non-DED (31.0%, 113/365 individuals), whereas 252 participants were diagnosed to have DED (69.0%, 252/365 individuals). The mean Japanese version of OSDI (J-OSDI) total score^23,24^ was 31.2 ± 28.8 points, and participants with DED had significantly higher J-OSDI total score than non-DED participants (40.8 ± 19.3 points *vs.* 9.8 ± 13.1 points, *P* < 0.001). The mean TFBUT was 1.7 ± 1.5 s, and participants with DED showed significantly lower TFBUT than non-DED participants (1.5 ± 0.8 s *vs*. 2.3 ± 2.4 s, *P* < 0.001). The mean corneal fluorescein staining (CFS) score was 2.3 ± 2.0 points, and participants with DED had significantly higher CFS than non-DED participants (2.5 ± 2.6 points *vs*. 1.9 ± 2.4 points, *P* = 0.031). The Schirmer test I value was not significantly different between the groups. The mean MBI was 11.3 ± 10.6 s, and participants with DED had significantly shorter MBI than non-DED participants (10.2 ± 2.3 s *vs*. 13.9 ± 7.8 s, *P* < 0.001).

**Table 1:** Characteristics of the study participants.

Classification	Non-DED	DED	*P* value	Total
characteristics	n = 113	n = 252		N = 365
Age, year ± SD	59.8 ± 18.2	60.7 ± 15.4	0.648	60.4 ± 16.3
Female, number (%)	91 (80.5)	220 (87.3)	0.092	311 (85.2)
BCVA, LogMAR ± SD	− 0.021 ± 0.11	− 0.022 ± 0.11	0.934	− 0.021 ± 0.11
IOP, mmHg ± SD	13.9 ± 2.8	14.0 ± 2.7	0.905	14.0 ± 2.8
J-OSDI total score, 0–100 ± SD	9.8 ± 13.1	40.8 ± 19.3	< 0.001	31.2 ± 28.8
TFBUT, s ± SD	2.3 ± 2.4	1.5 ± 0.8	< 0.001	1.7 ± 1.5
CFS score, 0–9 ± SD	1.9 ± 2.4	2.5 ± 2.6	0.031	2.3 ± 2.0
Schirmer I, mm ± SD	6.6 ± 7.7	5.6 ± 6.0	0.195	5.9 ± 6.6
MBI, s ± SD	13.9 ± 7.8	10.2 ± 2.3	< 0.001	11.3 ± 10.6

P values were estimated using the *t* test for continuous variables and χ^2^ test for categorical variables.

*DED* dry eye disease, *BCVA* best-corrected visual acuity, *IOP* intraocular pressure, *J-OSDI* Japanese version of Ocular Surface Disease Index, *TFBUT* tear film breakup time, *CFS* corneal fluorescein staining, *MBI* maximum blink interval, *SD* standard deviation.

### Discriminant validity of the characteristics based on the MBI with J-OSDI and TFBUT with J-OSDI classifications

Table 2 shows the comparison of the characteristics and the values of DED examinations based on the MBI with J-OSDI and TFBUT with J-OSDI classifications. The characteristics including age, female rate, best-corrected visual acuity, and intraocular pressure were not significantly different between the MBI with J-OSDI and TFBUT with J-OSDI groups. In the non-DED group, the J-OSDI total score was significantly increased in the MBI with J-OSDI group compared with the TFBUT with J-OSDI group (18.2 ± 19.1 *vs*. 9.8 ± 13.1, *P* < 0.001). The values of TFBUT, CFS, and Schirmer test I were not significantly different between the MBI with J-OSDI and TFBUT with J-OSDI groups. MBI was significantly decreased in the MBI with J-OSDI group compared with the TFBUT with J-OSDI group (7.4 ± 2.7 s *vs*. 10.2 ± 2.3 s, *P* < 0.001).

**Table 2:** Comparison of the participants’ characteristics between MBI with J-OSDI and TFBUT with J-OSDI classifications.

Classification	Non-DED			DED		
Methodology	TFBUT + J-OSDI	MBI + J-OSDI	*P* value	TFBUT + J-OSDI	MBI + J-OSDI	*P* value
Characteristics	n = 113	n = 167		n = 252	n = 198	
Age, years ± SD	59.8 ± 18.2	61.0 ± 17.3	0.592	60.7 ± 15.4	60.0 ± 15.5	0.614
Female, number (%)	91 (80.5)	137 (82.0)	0.877	220 (87.3)	174 (87.9)	0.887
BCVA, LogMAR ± SD	− 0.021 ± 0.11	− 0.011 ± 0.12	0.485	− 0.022 ± 0.11	− 0.030 ± 0.11	0.419
IOP, mmHg ± SD	13.9 ± 2.8	14.1 ± 2.8	0.695	14.0 ± 2.7	13.9 ± 2.6	0.681
J-OSDI total score, 0–100 ± SD	9.8 ± 13.1	18.2 ± 19.1	< 0.001	40.8 ± 19.3	42.1 ± 19.6	0.464
TFBUT, s ± SD	2.3 ± 2.4	2.1 ± 2.0	0.456	1.5 ± 0.8	1.4 ± 0.9	0.475
CFS score, 0–9 ± SD	1.9 ± 2.4	2.2 ± 2.5	0.266	2.5 ± 2.6	2.4 ± 2.6	0.653
Schirmer I, mm ± SD	6.6 ± 7.7	6.4 ± 7.3	0.838	5.6 ± 6.0	5.5 ± 5.8	0.851
MBI, s ± SD	13.9 ± 7.8	16.0 ± 7.3	0.023	10.2 ± 2.3	7.4 ± 2.7	< 0.001

P values were estimated using the *t* test for continuous variables and χ^2^ test for categorical variables.

*DED* dry eye disease, *BCVA* best-corrected visual acuity, *IOP* intraocular pressure, *J-OSDI* Japanese version of Ocular Surface Disease Index, *TFBUT* tear film breakup time, *CFS* corneal fluorescein staining, *MBI* maximum blink interval, *SD* standard deviation.

### Concurrent validity between MBI, J-OSDI, and dry eye examinations

Concurrent validity was assessed by calculating the correlations between MBI, J-OSDI, and dry eye examinations using the Pearson correlation test (Table 3). MBI was negatively correlated with J-OSDI total score (r = − 0.235, *P* < 0.001) and CFS (r = − 0.137, *P* = 0.009), whereas MBI was positively correlated with TFBUT (r = 0.430, *P* < 0.001). TFBUT was negatively correlated with CFS (r = − 0.301, *P* < 0.001), whereas TFBUT was positively correlated with Schirmer test I (r = 0.176, *P* = 0.001). CFS was negatively correlated with Schirmer test I (r = − 0.212, *P* < 0.001).

**Table 3:** Correlation between the MBI and other dye eye examinations.

Dry eye examinations	MBI	J-OSDI	TFBUT	CFS	Schirmer I
MBI	1.000				
J-OSDI	− 0.235***	1.000			
TFBUT	0.430***	− 0.056	1.000		
CFS	− 0.137**	− 0.013	− 0.301***	1.000	
Schirmer I	0.045	− 0.074	0.176**	− 0.212***	1.000

Pearson rank correlation coefficient was used to determine the correlations between the MBI and various dry eye examinations.

**P < 0.01, and ***P < 0.001.

*MBI* maximum blink interval, *J-OSDI* Japanese version of Ocular Surface Disease Index, *TFBUT* tear film breakup time, *CFS* corneal fluorescein staining.

### Test–retest reliability of MBI

The reliability of J-OSDI was confirmed by a previous study^23^. Therefore, the test–retest reliability of MBI was assessed by calculating the intraclass correlation coefficient (ICC) value from the first and second entries. The test–retest reliability of MBI was evaluated in 279 participants, with a median (interquartile range) period of 112 (63–182) days between the test and retest. The ICC value was 0.700 (95% confidence interval [CI] 0.65–0.75) for the MBI.

### Receiver operating characteristic curves for DED detection

**Figure 1:**
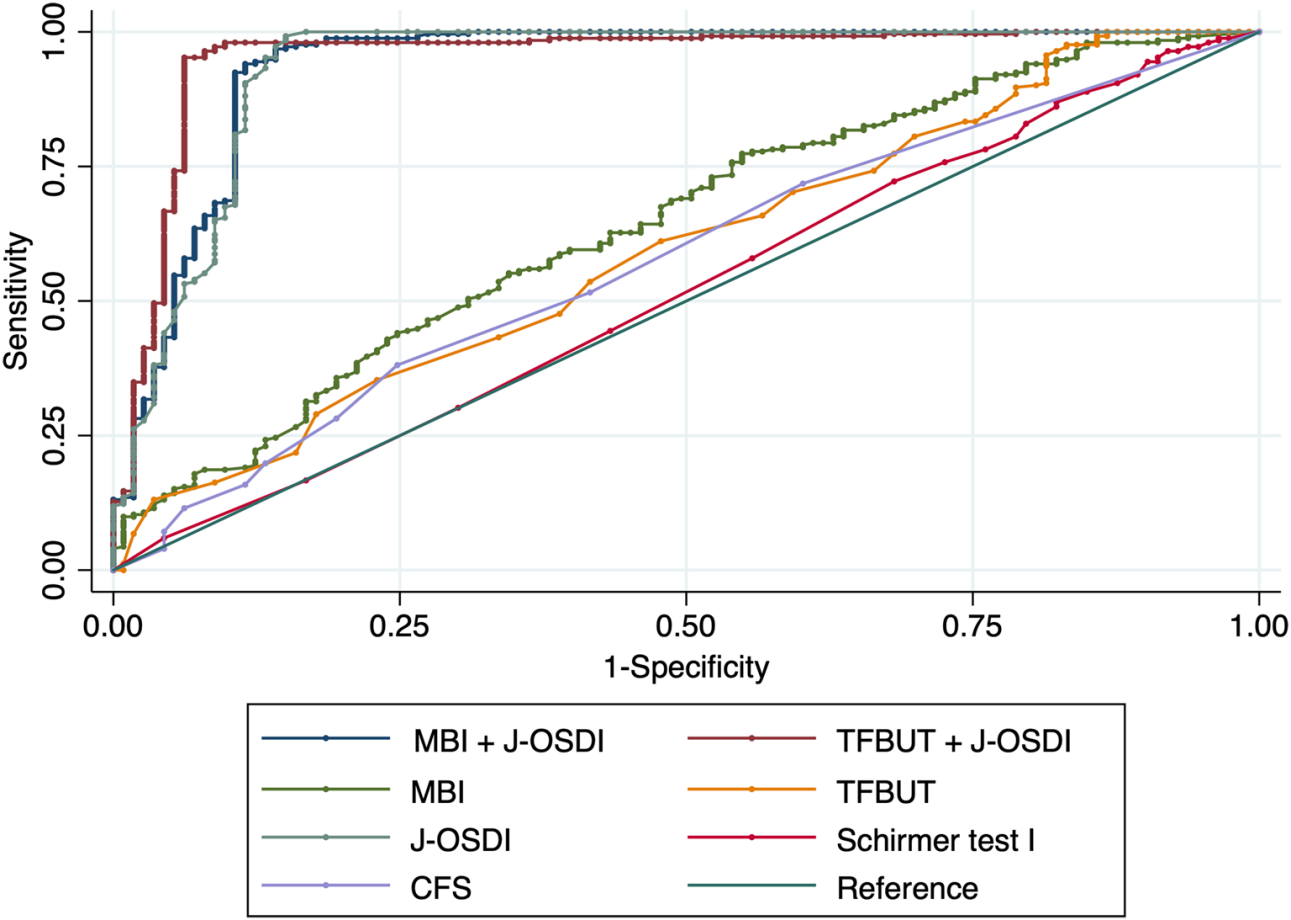
Receiver operating characteristic (ROC) curve for the detection of dry eye disease (DED). The ROC curve for the detection of non-DED or DED group classified by the Asia Dry Eye Society diagnostic criteria using dry eye examinations. *ROC* receiver operating characteristic curve, *DED* dry eye disease, *J-OSDI* Japanese version of the Ocular Surface Disease Index, *TFBUT* tear film breakup time, *CFS* corneal fluorescein staining, *MBI* maximum blink interval.

Figure 1 presents the receiver operating characteristic (ROC) curve for the detection of DED classified by the ADES diagnostic criteria using TFBUT with J-OSDI, MBI with J-OSDI, and each dry eye examination. The area under the curves (AUC) is shown in Table 4. The highest AUC was found for TFBUT with J-OSDI (0.954, 95% CI 0.925–0.983), followed by MBI with J-OSDI (0.938, 95% CI 0.904–0.971), J-OSDI (0.935, 95% CI 0.900–0.970), MBI (0.643, 95% CI 0.900–0.970), TFBUT (0.595, 95% CI 0.532–0.658), CFS (0.578, 95% CI 0.516–0.639), and Schirmer test I (0.517, 95% CI 0.452–0.582).

**Table 4:** AUC values determined by receiver operating characteristic curves to identify dry eye disease.

	AUC	SE	95% CI	
TFBUT + J-OSDI	0.954	0.015	0.925	0.983
MBI + J-OSDI	0.938	0.017	0.904	0.971
J-OSDI	0.935	0.018	0.900	0.970
MBI	0.643	0.031	0.581	0.704
TFBUT	0.595	0.032	0.532	0.658
CFS	0.578	0.031	0.516	0.639
Schirmer I	0.517	0.033	0.452	0.582

*TFBUT *tear film breakup time, *J-OSDI* Japanese version of Ocular Surface Disease Index, *MBI* maximum blink interval, *CFS* corneal fluorescein staining, *AUC* area under the ROC curve, *SE* standard error, *95% CI* 95% confidence interval.

### Precision rate detected between MBI with J-OSDI

Table 5 shows the precision rate diagnosed using MBI with J-OSDI. The positive predictive value was 96.0% (190/198 individuals), and the negative predictive value was 37.1% (62/167 individuals). The sensitivity and specificity were 75.4% (190/252 individuals) and 92.9% (105/113 individuals), respectively.

**Table 5:** Precision rate detected between MBI with J-OSDI and TFBUT with J-OSDI.

		TFBUT + J-OSDI		
		DED	Non-DED	
MBI + J-OSDI	DED	190	8	198
	Non-DED	62	105	167
		252	113	365

*MBI* maximum blink interval, *J-OSDI* Japanese version of Ocular Surface Disease Index, *TFBUT* tear film breakup time, *DED* dry eye disease.

### Individual positive signs and correlation of dry eye examination

**Figure 2:**
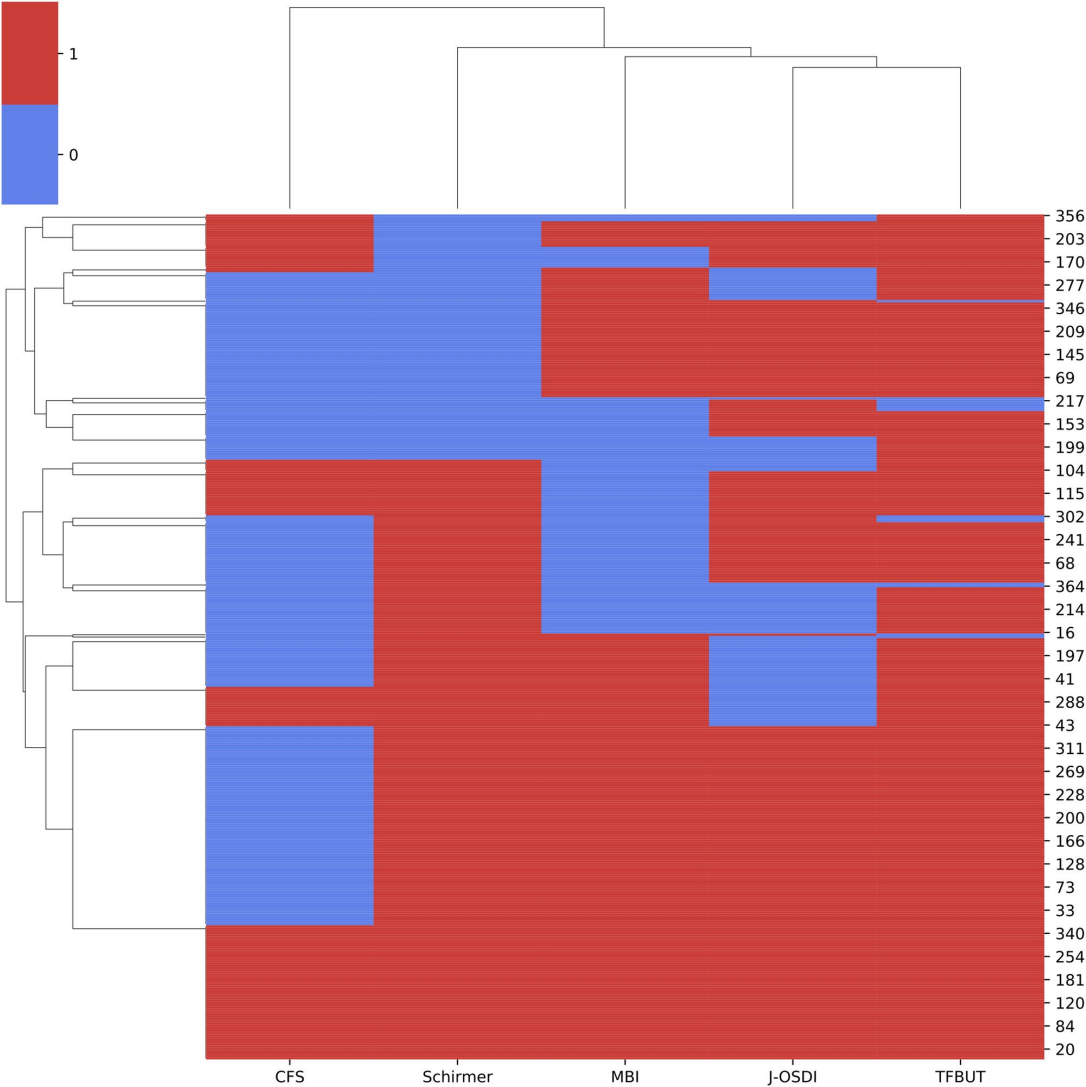
Individual positive signs of dry eye examinations using a clustered heatmap with dendrograms. Heatmap with clustering of the dry eye examinations of the individual participants. *CFS* corneal fluorescein staining, *MBI* maximum blink interval, *J-OSDI* Japanese version of Ocular Surface Disease Index, *TFBUT* tear film breakup time.

Figure 2 shows the individual positive sign of dry eye examinations using clustered heatmap with dendrograms. The heatmap represents individual positive signs between the examinations, indicating that the positive signs of MBI and TFBUT have some discrepancy. The dendrogram visualized the correlation of the dry eye examinations; J-OSDI, TFBUT, and MBI are clustered together, and CFS and Schirmer test I are clustered far.

## Discussion

Due to the importance of vision and acuity on the rapidly expanding digital society, a self-assessment and -management tool on DED is becoming more crucial for one’s work productivity. In this research, we investigated the simultaneous utilization of MBI and J-OSDI as a diagnostic tool for DED with results that are on par with the already-established OSDI and TFBUT tests. As MBI and J-OSDI are both non-invasive, simple methods of testing DED, their combinatory usage might be a great tool for healthcare providers as well as useful for individuals with undiagnosed DED during initial triage. The ease of use that this new test sequence offers align well with the pillars of P4 medicine—a medical model that advocates for the predictive, preventative, personalized, and participatory aspect of medicine. Such non-invasive tests that minimally affect one’s daily activities could contribute in early diagnosis and self-management^25^.

DED is a multifactorial disease, of which its causative factors are mainly divided into three categories, namely, environmental, host, and lifestyle factors^1^. However, in most cases, a curative option for dry eye patients is not available, and patients are limited with treatments to halt disease progression. As such, early diagnosis and prevention of DED becomes essential in combatting the societal loss caused by DED. As shown in Fig. 1 and Table 4, the diagnostic capabilities of the concurrent MBI and J-OSDI testing showed results comparable to the traditional TFBUT/J-OSDI testing based on ADES standards. This is likely due to the strong correlation that MBI showed with the two exams that are key in diagnosing DED. However, we noticed that these diagnostic tests might have a lower sensitivity (true negative rate) as shown in Table 2. In the non-DED cohort diagnosed using MBI/J-OSDI, the J-OSDI scores were higher than the diagnostic criteria of ≥ 13. This is also observed in the clustered heatmap with dendrograms (Fig. 2), where certain MBI-negative individuals showed positive TFBUT test results. As a result, the TFBUT-positive, but MBI-negative, individuals might have increased the J-OSDI value in the MBI/J-OSDI non-DED cohort. Nonetheless, the specificity (true positive rate) was comparable in both test methods when comparing the DED cohort, as well as specific metrics, including J-OSDI, TFBUT, CFS, and Schirmer test I results. No statistically significant difference was observed between MBI/J-OSDI and TFBUT/J-OSDI from the above metrics, and the 92.9% specificity indicates that a positive result is highly suggestive of DED. Taking advantage of their non-invasiveness and simplicity, MBI and J-OSDI testing holds promise in improving early diagnosis of DED through frequent longitudinal assessments.

The observed ROC curve for numerous diagnostic tests and their combinatory usage for DED revealed that the concurrent usage of MBI/J-OSDI significantly improved the AUC compared with that of MBI alone (Fig. 2). However, J-OSDI alone showed a high AUC, and the synergistic effect of MBI and TFBUT with J-OSDI was minimal. Considering that the average TFBUT of healthy individuals is approximately 7.6–9.1 s^26,27^, the sampling bias stemming from the lower-than-average TFBUT (mean = 1.7 s; lower than TFBUT cut-off of 5 s) and MBI in a DED intensive clinic could have caused the AUC of J-OSDI alone to be significantly higher than that of TFBUT or MBI alone.

The unmet medical need for an objective, non-invasive, and reproducible biomarker for DED has long been a task for clinicians who sought to improve the early diagnosis of DED for better long-term outcomes^28^. TFBUT, although often an important tool to assess the characteristics of one’s ocular surface, requires direct application of fluorescein on the ocular surface. The invasive nature of this exam is far from ideal, and low test reproducibility and tear stability disruption by the fluorescein stain itself have been reported^29–31^. Conversely, MBI and J-OSDI are both non-invasive tests, having great potential in self-diagnosis and self-management on a digital platform through online examinations. In this research, the test validity and reliability of MBI and J-OSDI were explored and confirmed. The concurrent usage of MBI and J-OSDI showed a sensitivity of 75.4%, specificity of 92.9%, positive predictive value of 96.0%, and negative predictive value of 62.9% on the diagnosis of DED.

A report on the test performance of online-based blink examination—where the participant was asked to perform non-forceful blinks, followed by a cease of blinking until discomfort with a timer—and OSDI questionnaires on DED diagnosis showed similar results as our MBI/J-OSDI results (sensitivity = 71%, specificity = 90%)^32^. Another report on J-OSDI-related apps and questionnaires also showed matching test results^4^, which leaves the validity and reliability of MBI in a remote setting as the last remaining portion to assess the utilization of MBI/J-OSDI in a mobile health environment for DED diagnosis. Mobile health devices, including smartphones and smartwatches, are aptly positioned for a continuous, longitudinal evaluation of one’s health conditions without the subjects leaving their daily activities^25^. Additionally, the now-commonplace biosignal sensors in mobile devices are further accelerating the revolution of the traditional facility-based healthcare paradigm toward a participatory, day-to-day healthcare within one’s daily life.

This study has several limitations. First, it might contain a degree of selection bias, as this study was conducted in a dry-eye-specific clinic at a single university hospital in Tokyo, Japan. Second, there were more female participants, likely due to the higher prevalence of DED among females^33^. In the future, a multicentered study with subjects selected from the general patient population should be considered. Third, socioeconomic status, educational level, cultural background data, and important unmeasured DED-related factors—including the use of systemic medications, depression, and anxiety—were not collected in this study. Fourth, seasonal effects were not examined due to the cross-sectional design of the study. Finally, this study was designed to investigate the diagnostic ability of MBI with J-OSDI as a simple and non-invasive screening method for DED detection. Thus, rose bengal staining scores, tear osmolality, meibomian gland dysfunction assessments, and corneal sensations were not investigated in this study. Despite these limitations, the diagnostic ability of MBI with J-OSDI was verified, and the satisfactory reliability and validity for DED detection could be useful for clinicians who seek minimally invasive diagnostic tools, as well as for the field of P4 medicine.

In this study, the capacity of MBI and J-OSDI on assessing DED suggests a novel, non-invasive route in the diagnosis of DED. Owing to the rapidly growing digital dependence of societies worldwide, the prevalence of DED is expected to increase, which underscores the importance of prevention and self-management at an early stage. With the potential observed in the MBI/J-OSDI test as a mobile examination, its implications in the epidemiology of DED prevention and management seem compelling in a global scale.

## Methods

### Study design and participants

This cross-sectional observational study included 365 patients recruited between September 2017 and December 2019 at the Department of Ophthalmology, Juntendo University Hospital, Tokyo, Japan. All participants gave written informed consent for the data to be used for research purposes. The clinical study was approved by the independent ethics committee of Juntendo University Hospital and adhered to the tenets of the Declaration of Helsinki.

### Exclusion criteria

We excluded patients with a history of eye lid disorder, ptosis, mental disease, Parkinson disease, and any other disease that affects blinking according to a previous study^21^.

### Dry eye disease diagnosis and classification

Both eyes in all patients underwent complete ophthalmic evaluation, including measurement of best-corrected visual acuity, intraocular pressure, and assessment of subjective symptoms using the J-OSDI^23^. Dry eye examinations including TFBUT, CFS for kerato-conjunctival vital staining, and Schirmer test I for reflex tear production were assessed in both eyes. As blinking is affected by both eye conditions via the corneal reflex^34^, the worst TFBUT and Schirmer test I value data were used, whereas higher values of CFS were used in this study. DED and non-DED were diagnosed using the 2016 ADES criteria^14^. The 2016 criteria make a diagnosis of DED with two positive items, specifically positive subjective symptoms and decreased TFBUT (≤ 5 s).

### Environmental conditions

Temperature and humidity of the examination room were controlled at 26 °C in the summer and 24 °C in the winter and 50% relative humidity, according to the Guideline for Design and Operation of Hospital HVAC Systems established by the Healthcare Engineering Association of Japan Standard^35^.

### Subjective symptom assessment using the J-OSDI

Subjective symptoms were assessed by interviewing the participants. The OSDI questionnaire is a 12-item instrument that was initially developed in 1997^36^. J-OSDI was validated by Inomata-Midorikawa et al.^23^ It was created to assess the subjective symptoms of DED and the effects of DED on vision-related activities of daily living^22,37^. The J-OSDI total score ranges from 0 to 100 points and is obtained by multiplying the total score of all the questions by 25 and dividing the result by the number of valid answers. The J-OSDI total score can be used to classify the respondent’s dry eye symptoms as normal (0–12 points), mild (13–22 points), moderate (23–32 points), or severe (33–100 points)^22,38,39^.

### Clinical assessments for DED

TFBUT and CFS were assessed with fluorescein sodium (Fluorescence Ocular Examination Test Paper, Ayumi Pharmaceutical Co., Tokyo, Japan) staining. TFBUT, CFS, MBI measurements, and subsequently Schirmer test I were evaluated.

### TFBUT

TFBUT was measured using a fluorescein dye according to the standard method^14^. To minimize the effect of the test strip on tear volume and TFBUT, a small quantity of the dye was administered with a wetted fluorescein strip. After the dye was instilled, the subject was instructed to blink three times to ensure adequate mixing of the dye with the tears. The time interval between the last blink and the appearance of the first dark spot on the cornea was measured with a stopwatch. The mean value of three measurements was used. The cutoff value of TFBUT ≤ 5 s was used to diagnose DED^14^.

### Kerato-conjunctival vital staining (CFS)

CFS was graded according to the van Bijsterveld grading system^40^, dividing the ocular surface into three zones: nasal bulbar conjunctiva, temporal bulbar conjunctiva, and cornea. Each zone was evaluated on a scale of 0–3, with 0 indicating no staining and 3 indicating confluent staining. The maximum possible score is 9.

### MBI

MBI is defined as the length of time that the participants could keep the eye open before blinking during each trial^21^. According to a previous study^21^, MBI was measured twice by a stopwatch under a light microscope without light. MBI was recorded as 30 s if it exceeded 30 s. The cutoff value of MBI ≤ 12.4 s was used as a positive sign for DED^14^.

### Schirmer test I

The Schirmer test I was performed without topical anesthesia after the completion of all other examinations. Schirmer test strips (Ayumi Pharmaceutical Co., Tokyo, Japan) were placed at the outer one-third of the temporal lower conjunctival fornix for 5 min. The strips were then removed, and the length of dampened filter paper (in mm) was recorded.

### Reliability

Test–retest reliability of MBI was evaluated by calculating the ICC values from the first and second entries. An ICC value of 0.70 was considered acceptable for test–retest reliability^41^.

### Validity

Discriminant validity was evaluated by comparing the non-DED and DED groups based on the MBI with J-OSDI and TFBUT with J-OSDI classifications. Concurrent validity was assessed by calculating the correlations (Pearson coefficients) between the J-OSDI total score and MBI; or other dry eye assessments including TFBUT, CFS, and Schirmer test I values.

### Statistical analyses

To compare the characteristics of the study participants, *t* test was used for continuous variables and χ^2^ test was used for categorical variables. ROC analysis was conducted to examine the diagnostic efficacy of MBI for DED. The ROC curve was plotted by computing the sensitivity and specificity using each symmetric value of the rating variable as a possible cutoff point. A point was plotted on the graph for each of the cutoff points; these plotted points were joined by straight lines to form the ROC curve, and the AUC was estimated using the trapezoidal rule. Data are presented as means ± standard deviations (SDs) or proportions. Statistical analyses were performed using STATA version 15 (Stata Corp, Texas, USA). *P* < 0.05 was considered significant.

## Data Availability

All data generated or analyzed during this study are included in this published article.
